# Lessons Learned from a Decade of Investigations of Shiga Toxin–Producing *Escherichia coli* Outbreaks Linked to Leafy Greens, United States and Canada

**DOI:** 10.3201/eid2610.191418

**Published:** 2020-10

**Authors:** Katherine E. Marshall, April Hexemer, Sharon L. Seelman, Marianne K. Fatica, Tyann Blessington, Maha Hajmeer, Hannah Kisselburgh, Robin Atkinson, Kristin Hill, Davendra Sharma, Michael Needham, Vi Peralta, Jeffrey Higa, Karen Blickenstaff, Ian T. Williams, Michael A. Jhung, Matthew Wise, Laura Gieraltowski

**Affiliations:** Centers for Disease Control and Prevention, Atlanta, Georgia, USA (K.E. Marshall, H. Kisselburgh, I.T. Williams, M.A. Jhung, M. Wise, L. Gieraltowski);; Public Health Agency of Canada, Ottawa, Ontario, Canada (A. Hexemer);; Food and Drug Administration, College Park, Maryland, USA (S.L. Seelman, M.K. Fatica, T. Blessington, K. Blickenstaff);; California Department of Public Health, Sacramento, California, USA (M. Hajmeer, M. Needham);; California Department of Public Health, Richmond, California, USA (V. Peralta);; California Department of Public Health, Los Angeles, California, USA (J. Higa);; Canada Food Inspection Agency, Ottawa (R. Atkinson, K. Hill, D. Sharma)

**Keywords:** Shiga toxin–producing Escherichia coli, disease outbreaks, leafy greens, vegetables, bacteria, United States, Canada, food safety, E. coli, enteric infections, STEC

## Abstract

Shiga toxin–producing *Escherichia coli* (STEC) cause substantial and costly illnesses. Leafy greens are the second most common source of foodborne STEC O157 outbreaks. We examined STEC outbreaks linked to leafy greens during 2009–2018 in the United States and Canada. We identified 40 outbreaks, 1,212 illnesses, 77 cases of hemolytic uremic syndrome, and 8 deaths. More outbreaks were linked to romaine lettuce (54%) than to any other type of leafy green. More outbreaks occurred in the fall (45%) and spring (28%) than in other seasons. Barriers in epidemiologic and traceback investigations complicated identification of the ultimate outbreak source. Research on the seasonality of leafy green outbreaks and vulnerability to STEC contamination and bacterial survival dynamics by leafy green type are warranted. Improvements in traceability of leafy greens are also needed. Federal and state health partners, researchers, the leafy green industry, and retailers can work together on interventions to reduce STEC contamination.

## Abstract 2

This analysis reveals patterns that may inform future prevention strategies.

Shiga toxin–producing *Escherichia coli* (STEC) cause an estimated 265,000 illnesses (*1*) and cost $280 million (*2*) annually in the United States. STEC infection can occur through exposure to contaminated food, water, or the environment or contact with infected animals or humans. STEC are broadly categorized by serogroup: STEC O157 and non-O157 STECs (all other serogroups). Infection with STEC O157, although less common than those caused by non-O157 STECs, can be severe. Persons infected with STEC O157 are more likely to be hospitalized and develop hemolytic uremic syndrome (HUS) more frequently than those infected with non-–O157 STECs (*3*).

In the United States, STEC O157 outbreaks were linked to contaminated leafy greens in 1995 and non-O157 STEC outbreaks in 2010 (*4*–*6*). In Canada, STEC O157 outbreaks have been linked to leafy greens since 2012 (Public Health Agency of Canada [PHAC], unpub. data). Leafy greens are the second most common source of foodborne STEC O157 outbreaks in both countries, after ground beef (*4*,*5*) (A. Hexemer, unpub. data). Many animals can be STEC hosts, but ruminants, primarily cattle, are considered the major reservoir (*7*–*10*). STEC shed from cattle and wild animals can directly contaminate leafy greens or indirectly contaminate them through irrigation water, runoff, or dust containing feces (*8*,*11*–*13*).

Most US-produced leafy greens (98%) are grown in California and Arizona (*14*). Leafy greens consumed in the United States are grown principally in the desert regions of California, Arizona, and Mexico in the winter months (November–March), and in the central coastal regions of California in the spring, summer, and fall months (April–October) (*15*). Most leafy greens consumed in Canada are imported from the United States (D. Burgoyne, Canadian Food Inspection Agency, pers. comm., 2019 May 31).

We reviewed epidemiologic, laboratory, and traceback data from STEC O157 and non–O157 outbreaks in the United States and Canada linked to leafy greens during 2009–2018. We summarize epidemiologic findings, describe barriers to solving outbreaks, and identify research needs to prevent future leafy green outbreaks.

## Methods

We collected data on STEC O157 and non–O157 outbreaks that were linked to leafy greens during 2009–2018 from the following sources: Centers for Disease Control and Prevention (CDC) Foodborne Disease Outbreak Surveillance System (FDOSS; 2009–2017 only); internal CDC and PHAC databases used to manage multistate outbreak investigations; and PulseNet, the national molecular subtyping network for foodborne disease surveillance (*16*). We defined an outbreak as >2 similar illnesses in persons with a common exposure. Outbreaks for which STEC was listed as the single causative pathogen, with >2 culture-confirmed cases of infection, and for which leafy greens were listed as a suspected or confirmed source, were included in this report. HUS was identified by physician diagnosis.

Local, state or provincial, and federal health officials assessed 3 types of evidence (epidemiologic, traceback, and microbiologic) to determine outbreak sources during an investigation. For epidemiologic evidence, health officials interviewed ill persons to gather detailed information on foods they ate, determine whether any foods were reported more frequently than expected (compared with the FoodNet population survey [*17*]), and determine whether persons ate food from the same point of sale (e.g., grocery stores, restaurants) or event (collectively defined as a subcluster). For traceback evidence, officials collected and evaluated records documenting the movement of foods to and from all points in a distribution chain (e.g., receipts, grocery store shopper cards, restaurant rewards numbers, invoices, bills of lading) to determine whether there was a common point of contamination from at least two distinct points of sale. The Canada Food Inspection Agency (CFIA) conducted traceback to points of importation, based on methodology employed in Canada (*18*). For microbiologic evidence, officials sampled foods and environments of restaurants, production facilities, or growing areas suspected to be the source of outbreaks and conducted microbiologic testing for the outbreak strain. Food sources were classified as suspected or confirmed outbreak vehicles based on the evidence collected during the investigation. For outbreaks linked to a single event or meal, only 1 type of evidence (epidemiologic, traceback, or microbiologic) was needed to be considered confirmed. For multistate outbreaks, or outbreaks during which ill persons reported exposures in multiple venues, vehicles were classified as suspected if only 1 type of evidence was identified and confirmed if >2 types of evidence were identified.

Outbreaks that occurred in both the United States and in Canada were counted as a single outbreak if they occurred at the same time and had the same outbreak strain. Outbreaks were classified by the state or province where ill persons were exposed to leafy greens.

We calculated the outbreak duration as the number of days between the first and last illness onset dates. We defined the outbreak investigation lag as the number of days between the first illness onset date and the date the coordinating agency began its investigation. We compared median outbreak size by vehicle status using the Kruskal-Wallis test and leafy green type by vehicle status using the Fisher exact test. We defined seasonality using the date of first illness onset for each outbreak and divided the year into 4 periods: spring (March–May), summer (June–August), fall (September–November), and winter (December–February). Outbreak vehicles were categorized as leafy greens according to the Interagency Food Safety Analytics Collaboration categorization schema (*19*). Outbreak strains were characterized using 2-enzyme pulsed-field gel electrophoresis.

CDC, FDA, PHAC, US state and local, and Canadian provincial health departments described outbreaks via press releases, Internet, Facebook, and Twitter posts to inform the public of measures they could take to protect themselves. Data on outbreak announcements were collected from CDC, FDA, and PHAC. Additional announcements may have been posted by state or provincial and local health departments but were not captured in this report.

## Results

### Epidemiology

**Figure 1:**
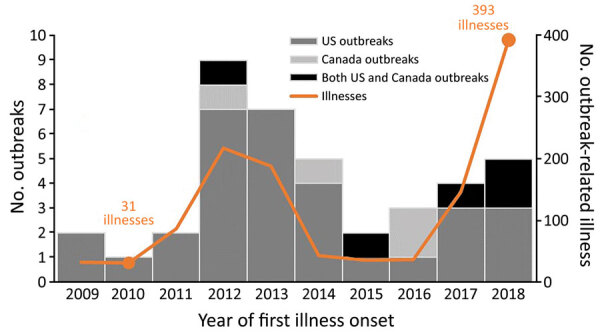
Number of Shiga toxin–producing *Escherichia coli* outbreaks (n = 40) linked to leafy greens in the United States, Canada, or both countries, and all outbreak-related illnesses (n = 1,212), by year of first illness onset, 2009–2018.

We identified 40 outbreaks of STEC infections during 2009–2018 with leafy greens as a confirmed (18 outbreaks) or suspected (22 outbreaks) source (Appendix Table). One additional STEC outbreak linked to leafy greens was excluded from analysis because it was caused by an ill food handler. Each year, 1–9 outbreaks occurred (Figure 1). Thirty-one outbreaks occurred in the United States only (22 multistate, 9 single state), 4 in Canada only (all multiprovince), and 5 in both Canada and the United States (4 multistate and multiprovince, 1 single state and multiprovince). These 40 outbreak investigations included 1,212 reported illnesses (1,146 laboratory-confirmed), 420 hospitalizations, 77 cases of HUS, and 8 deaths (Table 1). Ill persons ranged in age from <1 to 95 years (median 26); 63% were female. Outbreaks ranged from 3 to 248 (median 16) laboratory-confirmed illnesses; outbreaks with leafy greens as a confirmed source were larger than those with a suspected source (median 31 illnesses vs. 10 illnesses; p = 0.006).

**Table 1:** STEC outbreaks linked to contaminated leafy greens in the United States and Canada, 2009–2018*

Characteristic	US	Canada	Binational	All STEC
Outbreaks	31	4	5	40
Vehicle status				
Confirmed	14	1	3	18
Suspected	17	3	2	22
Serogroup				
STEC O157	24	4	4	32
Non-O157 STEC	7	0	0	7
Both	0	0	1	1
Total cases	677	65	470	1,212
Confirmed primary cases	621	65	460	1,146
Hospitalizations	203	26	191	420
Cases of HUS	35	4	38	77
Deaths	1	0	7	8

*HUS, hemolytic uremic syndrome; STEC, Shiga toxin–producing *Escherichia coli*.

Romaine lettuce was identified more often than any other type of leafy green as the outbreak source. Among the 29 (73%) STEC outbreaks with information on a specific leafy green type, 24 implicated a single type: 13 (54%) romaine, 4 (17%) spinach, 4 (17%) iceberg, and 1 (4%) each of cabbage, green leaf, and kale. (In 2015, the US investigation identified romaine lettuce as the outbreak source, and the Canadian investigation was not able to determine a specific type of leafy green. In 2017, the Canadian investigation linked an outbreak of STEC O157 to romaine lettuce, and the US investigation did not result in enough epidemiologic evidence to implicate a specific type of leafy greens. For the purposes of this article, the leafy green type was classified as unknown for these outbreaks.) Among the 24 outbreaks linked to a single lettuce type, 11 were confirmed, and romaine was more likely to be confirmed than any other leafy green type (10/13 vs. 1/11; p = 0.002). Five outbreaks were linked to multiple leafy green types: 3 romaine and iceberg, 1 butter and radicchio, and 1 spinach and spring mix (Table 2).

**Table 2:** STEC outbreaks linked to leafy greens by type of leafy green implicated, United States and Canada, 2009–2018*

Leafy green type	Outbreaks with information for type of leafy green†	Outbreaks with single known type of leafy green implicated	Outbreak-related illnesses attributed to outbreak with single type of implicated leafy green
Romaine	16 (40)	13 (54)	617 (84)	
Iceberg	7 (18)	4 (17)	54 (7)	
Spinach	5 (13)	4 (17)	32 (4)	
Cabbage	1 (3)	1 (4)	16 (2)	
Kale	1 (3)	1 (4)	7 (1)	
Green leaf	1 (3)	1 (4)	5 (0.7)	
Butter lettuce	1 (3)	0	0	
Radicchio	1 (3)	0	0	
Spring mix	1 (3)	0	0	
Unknown	11 (28)‡	0	0	
Total	40	24	731	

*Values are no. (%) except as indicated. STEC, Shiga toxin–producing *Escherichia coli* †More than 1 type of leafy green may have been reported for a given outbreak. ‡This includes two outbreaks that occurred in both the US and Canada. In 2015, the US investigation identified romaine lettuce as the outbreak source, and the Canadian investigation was not able to determine a specific type of leafy green. In 2017, the Canadian investigation linked an outbreak of STEC O157 to romaine lettuce, and the US investigation did not result in enough epidemiologic evidence to implicate a specific type of leafy green. For the purposes of this study, the leafy green type for these outbreaks was classified as unknown. For 1 outbreak, multiple leafy green types, including kale, spinach, and romaine, were reported and traced back but the leafy green type remained unknown.

**Figure 2:**
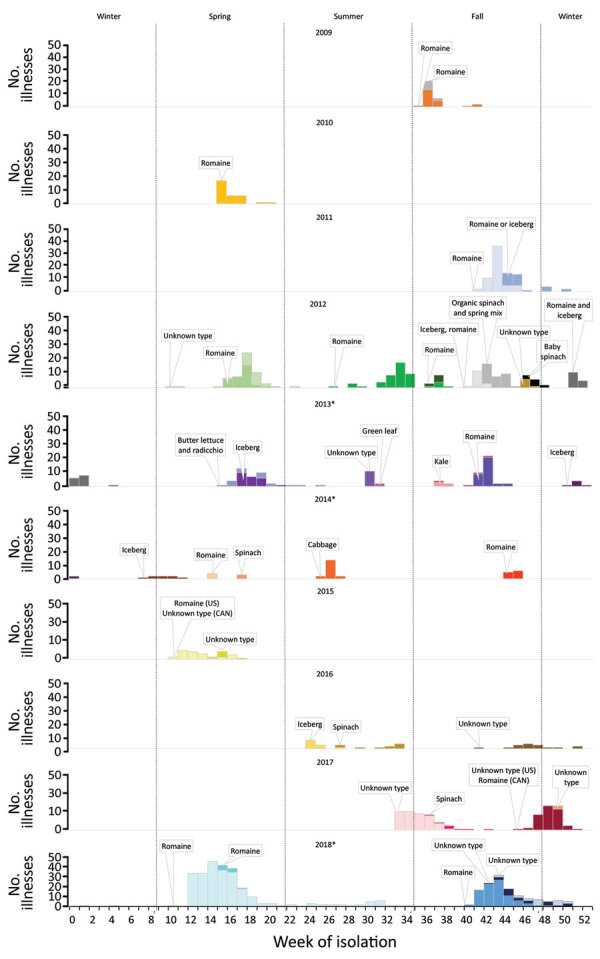
Outbreak-related Shiga toxin–producing *Escherichia coli* illnesses (n = 1,124) linked to leafy greens, by isolation date, outbreak, and vehicle, United States and Canada, 2009–2018. Data on isolation date were available for 1,124 (98%) of 1,146 laboratory-confirmed illnesses. Illness onset date was not systematically collected for each case. Each color represents a different outbreak. *During these years, each season started 1 week earlier.

**Figure 3:**
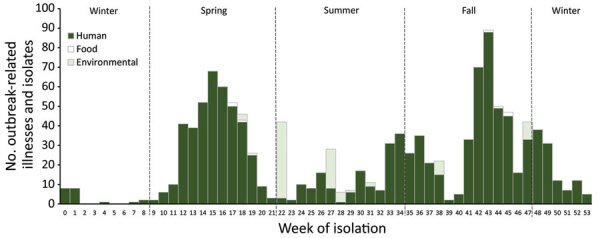
Outbreak-related Shiga toxin–producing *Escherichia coli* laboratory-confirmed illnesses (n = 1,124 illnesses for which information was available) and food (n = 8; spinach, romaine) and environmental isolates (n = 86; soil, water, sediment, scat) linked to leafy greens, by week of isolation, United States and Canada, 2009–2018.

More STEC outbreaks linked to leafy greens began during the fall (18, 45%) than spring (11, 28%), summer (7, 18%), or winter (4, 10%) (Figures 2, 3). More outbreaks began in October (9 outbreaks, 23%) and April (8 outbreaks, 20%) than any other month. The median outbreak duration was 21 days (range 1–162 days). The median investigation lag was 22 days.

### Environmental and Laboratory Testing

STEC O157 was the most common cause of leafy green STEC outbreaks. Among the 40 STEC outbreaks, 32 (80%) were caused by STEC O157; 3 by (8%) STEC O145; 2 (5%) by STEC O26; 1 (3%) each by serogroups STEC O111 and STEC O126; and 1 by both STEC O26 and STEC O157 (Appendix Table).

Of investigations with information, investigators found the outbreak strain in leafy greens in 2 outbreaks, and in the environment where greens were processed or grown in 4 outbreaks (Appendix Table). In 1 investigation, the outbreak strain was isolated from irrigation canal water samples collected upstream and downstream from a cattle concentrated animal feeding operation (CAFO) and in the area of several romaine farms identified during traceback (*20*). In a second investigation, the outbreak strain was isolated from sediment from a water reservoir on a romaine farm identified through traceback (*21*). In 2 other outbreak investigations, isolates collected during a separate project assessing STEC prevalence in California Central Coast watersheds were uploaded to PulseNet and matched the outbreak strains (*22*).

### Traceback

In the United States, traceback was conducted by FDA (15 outbreaks) and the California Department of Public Health (CDPH; 11 outbreaks). Some traceback investigations overlapped with multiple agencies investigating the same incident. Each traceback included 2–23 points of sale (median 4); 1–9 ill persons were associated with each point of sale. Points of sale were distributed across 1–12 states (median 2). When both FDA and CDPH conducted traceback for a multistate outbreak, FDA data were used to calculate the median. For some outbreaks, US and Canadian information was combined to determine a common source; data from Canada were removed from the US summary for these results.

CFIA conducted traceback for 7 of 9 outbreaks. For these 7 outbreaks, leafy greens were traced back from 2–30 points of service, 1–11 distributors/processors, multiple brands, and <21 suppliers. Two examples that highlight the complexity of traceback include an outbreak in 2012 linked to iceberg and romaine mix imported to Canada from the United States, which was mixed and packaged in 21 product combinations comprising 18 lots. A second outbreak in 2015 was not linked to a specific leafy green type, but multiple greens (kale, spinach, and romaine) were reported and traced back; investigators identified 53 potentially implicated products from 11 distributors. Most leafy greens were imported to Canada from the United States.

### Public Messaging and Product Action

Five (12.5%) of 40 outbreaks resulted in a food recall (Appendix Table). Recalled items included bagged shredded romaine, bagged spinach and spring mix, shredded iceberg and romaine, and ready-to-eat salads and sandwich wraps containing romaine. In a fall 2018 outbreak linked to romaine lettuce, potentially contaminated romaine lettuce was not recalled because it was no longer available for sale. However, the implicated firm voluntarily recalled other leafy greens and vegetables that came into contact with agricultural water from a reservoir with sediment that yielded the outbreak strain.

Nine (23%) leafy green STEC outbreaks were publicly announced by federal agencies, usually when there was an action that consumers could take to prevent illness (https://www.cdc.gov/foodsafety/outbreaks/investigating-outbreaks/communication/index.html). These actions included not eating recalled leafy greens (4 outbreaks) or not eating leafy greens grown in a specific region or county (2 outbreaks). Three outbreak postings did not advise consumers to take action around any specific leafy greens but informed the public of the investigation. Leafy greens were usually out of the supply chain (and therefore unavailable to consumers) by the time the investigation identified them as the outbreak source, minimizing the ongoing risk to the public and reducing the need for immediate public notification.

## Discussion

Over the past decade, multiple STEC outbreaks linked to leafy greens occurred in the United States and Canada, causing illness that was widespread and often severe. Most STEC outbreaks linked to leafy greens were caused by STEC O157, even though non-O157 STEC cause more sporadic US illnesses and are more frequently isolated from cattle (*10*); reasons for this discrepancy are unclear.

Despite year-round US leafy green production, 73% of STEC outbreaks linked to leafy greens began during the spring or fall. This seasonality was noted in a previous study of STEC O157 outbreaks (*5*); however, reasons for this seasonal pattern are unclear. Seasonal differences in consumption and production are one possible explanation. However, US data from 2007 (CDC FoodNet, unpub. data) and Canadian data from 2014–2015 (*23*) indicate that leafy green consumption changed little by month and did not show increases during the spring and fall. Data for leafy greens produced in 2009 showed some variation in domestic shipment volume by month but did not show an apparent increase in shipments in the spring and fall (*14*). Notably, the peak outbreak months in our report (October and April) coincided with the period when growers have historically used the short-term California Central Valley growing region to fill the gap in leafy green production between the California Central Coast and the desert regions of California, Arizona, and Mexico (*24*). We could not further assess any potential link between outbreak timing and harvest location because the movement of growing and harvesting operations varies by year and company, and there was a limited number of outbreaks during 2009–2018 for which growing locations were identified. Additional data on growing and harvesting practices, intrinsic factors of leafy greens that might make them susceptible to contamination, and the effect of climate or specific weather events during the growing seasons are needed to further assess seasonality of outbreaks.

Environmental assessments rarely occurred during investigations of leafy green outbreaks. Environmental assessments can occur only after epidemiologic and traceback investigations identify where implicated product is grown, processed, or distributed. Although they are resource intensive, these assessments can provide valuable insight into outbreak causes and identify possible areas to target for prevention efforts. Environmental assessments conducted during multiple leafy green investigations have suggested a possible link between product contamination and STEC contamination of nearby soil or water caused by cattle or wild pigs (*25*,*26*), dairy farms (*27*), or CAFOs (*28*). These findings build on 2 studies conducted in leafy green growing regions in California; one identified a higher prevalence of STEC O157 in a watershed near cattle (*29*), and another detected STEC O157 in beef cattle feces, mostly during dates in the spring and fall when leafy greens are typically grown, although the number of samples that yielded STEC O157 was small (*13*). Furthermore, some leafy green growing regions are home to large numbers of cattle: the Central Valley growing region (encompassing Fresno, Tulare, and Kings Counties) had >1.8 million head of cattle in 2016–2017, comprising nearly one third of all cattle in California (*30*). Conducting additional environmental assessments to better understand the relationship between cattle and leafy green growing locations, water, and the occurrence and timing of outbreaks may be beneficial.

More STEC outbreaks were linked to romaine than to any other type of leafy green, similar to an analysis of leafy greens–related incidents linked to California (*31*). More iceberg was harvested and available for purchase than romaine each year during 2009–2017, although romaine harvest and availability increased over time (*32*,*33*). The share of category dollars spent on iceberg and romaine was the same during 2012, higher for iceberg in 2013–2014, and higher for romaine in 2016–2017 (*34*). Together, these data suggest that even though romaine increased in popularity, it is unlikely that this alone explains why more STEC outbreaks in the past decade were linked to romaine than to any other leafy green. Because standard investigation questionnaires include questions about multiple leafy green types, such as spinach, iceberg, kale, and romaine (*35*), investigational bias toward romaine is unlikely. Romaine may have some characteristics that may make it more vulnerable to STEC contamination, including its shape and physiology (romaine is tall with loosely clumped leaves, open at the top; iceberg is smaller with compact leaves). Additional studies comparing the likelihood of STEC contamination and bacterial survival dynamics by leafy green type are warranted.

More than half of STEC outbreak investigations identified leafy greens as a suspected, rather than confirmed, source. Several characteristics of leafy green outbreaks make them inherently difficult to solve, and therefore challenging to implement timely interventions to reduce illness. The short shelf life of leafy greens (12–16 days) (*36*), the lag in identifying outbreaks (22 days), and the short duration of most outbreaks (21 days) all limit opportunities for investigators to interview ill persons in a timely fashion. This limitation can hamper hypothesis generation and limit opportunities to test leafy greens for contamination. Finally, because leafy greens, especially iceberg and romaine, are commonly consumed in the United States (*17*) and Canada (*23*), it can be difficult to show that they were eaten more often than expected by ill persons who are part of outbreaks. Establishing an epidemiologic link between cases and contaminated leafy greens often requires other corroborating pieces of evidence (e.g., brand or variety) to implicate a specific leafy green type. To help solve outbreaks, investigators have used successful strategies such as subcluster and purchase record analyses (*37*–*39*).

Traceback investigations for leafy green outbreaks are complex. First, product information from packaging is rarely available when an investigation begins. Therefore, ill persons are asked to remember crucial information needed to identify and trace leafy greens (e.g., purchase location and date, type/brand) instead of simply referring to an open package. Second, associating leafy greens at a point of sale location with a particular distribution lot can be challenging. Even though lot information may be available on the packaging for prepackaged leafy greens, points of sale may not record and track it after the packages are received. Investigators rely solely on records collected at each point in the distribution chain to determine the lots and source of a product, but they often lack the data elements needed to link lots of incoming shipments of products with lots of outgoing shipments. Finally, commingling of leafy greens from different farms throughout the distribution chain further complicates efforts to identify a single lot or source.

Complete, detailed records of transactions at each point along the fork-to-farm continuum are critical to accurately and quickly trace leafy greens during an outbreak investigation. Several strategies could increase the likelihood of success. Industry could assist by maintaining records that are consistently available, accurate, and complete. Retailers that sell leafy greens could consider developing systems to track lot and source information for leafy greens after they are received. Retailers may also wish to require producers be able to trace leafy greens and components of packaged mixes back to the farms from which they were harvested.

Several policies and recommendations were put into place before and after the study to improve the safety of leafy greens and prevent future outbreaks. In 2011, the US FDA Food Safety Modernization Act (FSMA) was signed into law (*40*). Under that law, in 2016, the Final Rule for Produce Safety went into effect, which established science-based minimum standards for the safe growing, harvesting, packing, and holding of US produce, including leafy greens. The first major compliance date for produce other than sprouts was in January 2018 (*41*). Routine regulatory inspections were set to begin in spring 2019, and compliance with agricultural water requirements were extended to become effective in 2022 (*42*). In 2007, the California and Arizona leafy greens industries each formed their own leafy greens products handler marketing agreement and enacted food safety recommendations (*43*) after a large 2006 STEC O157 outbreak linked to spinach (*25*). In response to the 2 large 2018 outbreaks, in 2019, California and Arizona modified their recommendations for leafy green growers, including increasing buffer zones between CAFOs and leafy green fields; requiring environmental assessments after severe weather events; requiring that all lot data be identified for products entering the marketplace; limiting or prohibiting the use of surface water for overhead irrigation of leafy greens before harvest; and requiring farmers to categorize sources of water, consider how it is applied to leafy greens, and test and sanitize it if needed (*44*–*47*). Future analyses should be conducted to assess the effect of these policies, recommendations, and any other implemented changes.

STEC outbreaks linked to leafy greens have continued to occur over the past decade. The combination of challenges investigators face during epidemiologic and traceback investigations of leafy greens make timely communication of actionable advice for consumers difficult. Despite challenges, results from leafy green outbreak investigations have led to changes in industry recommendations. However, knowledge gaps remain, including the drivers of the seasonality of leafy green outbreaks, and knowledge of why outbreaks are disproportionately linked to romaine lettuce. Investigators should work with federal and state health partners, the research community, the leafy green industry, and retailers to fill these knowledge gaps and collect additional information. Additional efforts should include identifying data points that would improve traceability of leafy greens during outbreaks. Collectively, these efforts can help inform prevention strategies to avoid or mitigate future outbreaks and lead to further changes in the way food is grown and processed, which could make leafy greens safer for the public to consume.

Appendix Additional information about the study of Shiga toxin–producing *Escherichia coli* outbreaks linked to leafy greens, United States and Canada.
