# Supplementary material for: Lessons Learned from a Decade of Investigations of Shiga Toxin–Producing Escherichia coli Outbreaks Linked to Leafy Greens, United States and Canada
Source: Emerg Infect Dis. 2020 Oct;26(10):2319–28. doi: 10.3201/eid2610.191418 (PMC7510726; doi:10.3201/eid2610.191418)
Supplement: Appendix — Additional information about the study of Shiga toxin–producing Escherichia coli outbreaks linked to leafy greens, United States and Canada. [file 19-1418-Techapp-s1.pdf]

Article DOI: https://doi.org/10.3201/eid2610.191418

Lessons Learned from a Decade of Investigations of Shiga Toxin-Producing *Escherichia coli* Outbreaks Linked to Leafy Greens, United States and Canada

Appendix

Appendix Table. Shiga toxin-producing *Escherichia coli* outbreaks linked to leafy greens in the United States and Canada, 2009–2018 (n = 40)*

First illness onset	Country	Distribution of cases	STEC serotype	No. illnesses	Median age (range)	No. hospitalizations	No. HUS cases	No. deaths	Vehicle	Vehicle status	Types of evidence	Recall?	Product recalled	Outbreak strain isolated from leafy greens?	Outbreak strain isolated from environment?
2009 Sep	United States	Multistate	O157:H7	22	22 (1–60)				Romaine lettuce	Confirmed	E, T	N			
2009 Sep	United States	Single state	O157:H7	10	29 (5–55)	1		0	Romaine lettuce	Suspected	E	N			
2010 Apr	United States	Multistate	O145	31	19 (1–31)	14	4	0	Romaine lettuce	Confirmed	E, T, M	Y	Bagged shredded romaine	Yes; unopened bag of shredded romaine at an institution	
2011 Oct	United States	Multistate	O157:H7	61	30 (1–93)	35	3	0	Romaine lettuce	Confirmed	E, T	N			
2011 Oct	United States	Multistate	O157:H7	26	30 (2–72)	5	0	0	Romaine or iceberg lettuce	Suspected	E	N			Yes; Salinas Valley Watershed, collected as part of a separate project. Testing of irrigation reservoir, retention pond, and drains all negative
2012 Mar	United States	Multistate	O157:H7	24	15 (<1–88)				Unknown leafy green type	Confirmed	E, T	N			
2012 Apr	United States and Canada	Single state and Multiprovince	O157:H7	35	32 (1–84)	13			Romaine lettuce	Confirmed	E, T	N			
2012 Jun	United States	Multistate	O157:H7	52	25 (2–77)				Romaine lettuce	Suspected	E	N			Yes; Salinas Valley Watershed, collected as part of a separate project.
2012 Sep	United States	Single state	O157:H7	9	33 (19–81)	7	0	0	Romaine lettuce	Suspected	E	N			
2012 Oct	United States	Multistate	O145	16	31 (2–78)	6	0	0	Romaine lettuce, iceberg lettuce	Suspected	E	N			

First illness onset	Country	Distribution of cases	STEC serotype	No. illnesses	Median age (range)	No. hospitalizations	No. HUS cases	No. deaths	Vehicle	Vehicle status	Types of evidence	Recall?	Product recalled	Outbreak strain isolated from leafy greens?	Outbreak strain isolated from environment?
2012 Oct	United States	Multistate	O157:H7	33	25 (4–66)	13	2	0	Spinach, spring mix	Confirmed	E, T, M	Y	Bagged spinach and spring mix	Yes: bags of organic spinach and spring mix collected from 4 ill persons' homes	
2012 Nov	United States	Single state	O157:H7	8	19 (18–21)	4	1	0	Unknown leafy green type	Confirmed	E, T	N			
2012 Nov	United States	Multistate	O157:NM (H-)	10	26 (14–71)			0	Spinach	Suspected	E	N			
2012 Dec	Canada	Multiprovince	O157	31	21 (1–83)	13	1	0	Romaine and iceberg lettuce	Confirmed	E, T	Y	Shredded iceberg and romaine lettuce		
2013 Apr	United States	Multistate	O157:H7	14	31 (2–88)	9	1	1	Butter lettuce, radicchio	Confirmed	E, T	N			
2013 Apr	United States	Multistate	O26	26	23 (3–81)	5		0	Iceberg lettuce	Suspected	E	N			
2013 Jul	United States	Single state	O157:H7	94	21 (1–69)	22	2	0	Unknown leafy green type	Confirmed	E, T	N			
2013 Jul	United States	Single state	O157:H7	5	66 (21–91)	5	4	0	Green leaf lettuce	Suspected	E	N			
2013 Sep	United States	Single state	O157:H7	7	63 (34–69)	5	2	0	Kale	Suspected	E	N			
2013 Oct	United States	Multistate	O157:H7	33	31 (4–78)	9	2	0	Romaine lettuce	Confirmed	E, T	Y	Ready-to-eat salads and sandwich wrap products		
2013 Dec	United States	Single state	O157:H7	9	27 (2–78)	8	4	0	Iceberg lettuce	Suspected	E	N			
2014 Feb	Canada	Multiprovince	O157:H7	8	21 (4–59)	4	1	0	Iceberg lettuce	Suspected	E	N			
2014 Apr	United States	Single state	O126	4	17 (15–17)	0	0	0	Romaine lettuce	Confirmed	E, T	N			
2014 Apr	United States	Multistate	O157:H7	4	19 (13–19)	1		0	Spinach	Suspected	E	N			
2014 Jun	United States	Multistate	O111	16	35 (16–84)	2		0	Cabbage	Confirmed	E, T	N			
2014 Nov	United States	Multistate	O157:H7	11	32 (20–70)	2	0	0	Romaine lettuce	Confirmed	E, T	N			
2015 Mar	United States and Canada	Multistate and multiprovince	O157:H7	29	24 (6–76)	12	1	0	Unknown leafy green type (Canada), romaine lettuce (United States)	Suspected	E	N			
2015 Apr	United States	Multistate	O145	7	18 (12–55)	5		0	Unknown leafy green type	Suspected	E	N			

First illness onset	Country	Distribution of cases	STEC serotype	No. illnesses	Median age (range)	No. hospitalizations	No. HUS cases	No. deaths	Vehicle	Vehicle status	Types of evidence	Recall?	Product recalled	Outbreak strain isolated from leafy greens?	Outbreak strain isolated from environment?
2016 Jun	United States	Multistate	O157:H7	11	25 (2–63)	4	2	0	Iceberg lettuce	Suspected	E	N			
2016 Jul	Canada	Multiprovince	O157:NM	10	21 (11–67)	2	0	0	Spinach	Suspected	E	N			
2016 Oct	Canada	Multiprovince	O157:H7	16	37 (4–94)	7	2	0	Unknown leafy green type	Suspected	E	N			
2017 Aug	United States	Multistate	O157:H7	69	29 (4–91)	18	2	0	Unknown leafy green type	Confirmed	E, T	N			
2017 Sep	United States	Multistate	O26	8	19 (15–51)	3	0	0	Spinach	Suspected	E	N			
2017 Nov	United States and Canada	Multistate and multiprovince	O157:H7	67	31 (1–95)	26	5	2	Unknown leafy green type (United States), romaine lettuce (Canada)	Suspected	E	N			
2017 Dec	United States	Single state	O157	3	18 (18–21)	3	0	0	Unknown leafy green type	Suspected	E	N			
2018 Mar	United States and Canada	Multistate and Multiprovince	O157:H7, O26	248	27 (1–93)	105	28	5	Romaine lettuce	Confirmed	E, T, M	N			Yes; irrigation canal water samples collected up and downstream from a concentrated animal feeding operation (CAFO) and in the area of several romaine farms identified during traceback
2018 Apr	United States	Multistate	O157:H7	10	28 (14–56)	5		0	Romaine lettuce	Confirmed	E, T	N			
2018 Oct	United States and Canada	Multistate and Multiprovince	O157:H7	91	34 (1–93)	35	4	0	Romaine lettuce	Confirmed	E, T, M	Y	Red leaf lettuce, green leaf lettuce, cauliflower		Yes; sediment from water reservoir on a romaine farm identified through traceback
2018 Oct	United States	Multistate	O157:H7	25	23 (2–79)	8	4	0	Unknown leafy green type	Suspected	E	N			
2018 Oct	United States	Multistate	O157:H7	19	43 (3–91)	4	2	0	Unknown leafy green type	Suspected	E	N			

*Information was missing if no number was provided. E, epidemiologic; HUS, hemolytic uremic syndrome; M, microbiological; STEC, Shiga toxin-producing *Escherichia coli;* T, traceback.
